# ADHD symptoms and learning behaviors in children with ASD without intellectual disability. A mediation analysis of executive functions

**DOI:** 10.1371/journal.pone.0207286

**Published:** 2018-11-14

**Authors:** Belen Rosello, Carmen Berenguer, Inmaculada Baixauli, Carla Colomer, Ana Miranda

**Affiliations:** 1 Department of Developmental and Educational Psychology, University of Valencia,Valencia, Spain; 2 Faculty of Psychology, Teaching and Education Sciences, Catholic University of Valencia "San Vicente Mártir", Valencia, Spain; 3 Department of Education, Universitat Jaume I, Castellón de la Plana, Spain; Liverpool John Moores University, UNITED KINGDOM

## Abstract

In spite of its importance for education, the relationship between learning behaviors (LB), attention deficit hyperactivity disorder symptoms (ADHD) and executive functioning (EF) in children with autism spectrum disorder (ASD) has hardly been explored. The first objective of the present study was to compare children with ASD without intellectual disability and children with typical development (TD) on ADHD symptoms and learning behaviors: Motivation/competence, attitude toward learning, persistence on the task, and strategy/flexibility. The second objective was to analyze the mediator role of behavioral regulation and metacognition components of EF between ADHD symptoms and learning behaviors in children with ASD. Participants were 89 children between 7 and 11 years old, 52 with ASD and 37 with TD, matched on age and intelligence. Their teachers filled out questionnaires assessing executive functioning as well as learning behaviors. Parents and teachers reported on inattention and hyperactivity/impulsivity behaviors. Compared to children with TD, children with ASD presented significantly more ADHD symptoms and poorer learning behaviors. In addition, there were significant mediation effects of the behavioral regulation index (BRI) and metacognition index (MI) of EF, indicating that both are part of the route through which ADHD symptoms impact to learning behaviors of children with ASD.

## Introduction

Autism spectrum disorder (ASD) is a neurodevelopmental disorder characterized by persistent difficulties in social communication and interaction and restrictive, repetitive, and stereotyped patterns of behavior, activities, and interests [1]. ASD has a strong genetic base with an estimated heritability of between 64% and 91% [2], and it follows a course remarkably stable over time across childhood [3]. Moreover, it is one of the most frequent neurodevelopmental disorders; its prevalence has multiplied by 4 in the past decade, reaching the current percentages of 1 in 68 children in the United States [4], with a greater presence in boys than girls, 3:1[5]. The greatest increase in ASD has occurred in the subgroup that does not present an intellectual handicap, referred to as “high-functioning autism” (HFA) with a prevalence of 16.8 per 1.000 (1/59) in children aged 8 [6,7]. Based on the development of cognitive and language skills, students with ASD without intellectual disability would be expected to achieve good academic results. However, longitudinal studies indicate that these factors do not predict academic success [8], so that many school children with ASD will need educational support programs while in school.

The body of literature on academic skills in children with autism is extensive, with the reviews highlighting the variability in the academic performance across the spectrum and in the different academic areas [9]. However, even at the extreme end of the intellectual dimension, in high-functioning autism there are difficulties in dealing with academic demands. Although many of these children present areas of strength in the cognitive and academic domain, their functioning is inconsistent. For example, they show problems in the comprehension of abstractions, metaphors, and other figures of speech, an excessive application of literal interpretations, or serious difficulties related to solving common problems in everyday life [10]. Deficits are especially evident on tasks that require conceptual and abstract learning, such as in maths, where 20% of children with high functioning autism show severe difficulties [11]. Reading comprehension is another area of difficulties for readers with autism [12]. They can understand stories and infer the emotions of characters in short narratives, but they have difficulties to question-answering about those same inferred emotions and on the type of demands this kind of task poses [13].

The understanding of the factors that can be related to the educational results of students with ASD is limited. Although the associations among IQ, academic progress, and response to students’ intervention is significant [14], the academic performance is also influenced by other factors related to personal and environmental characteristics. Many individuals with ASD perform significantly above or below the level predicted by their IQ [15], which reveals the need to broaden the focus by integrating modifiable factors that can contribute to academic success. Di Perna and Elliott [16] proposed a theoretical model of academic competence based on the construct of “academic enablers”, which are defined as “attitudes and behaviors that allow a student to participate in, and ultimately benefit from academic instruction in the classroom” (p.294). In addition to interpersonal skills, academic enablers include a series of behavioral skill areas referred to as competence motivation, control of attention, persistence, curiosity and flexibility. Children’s social and academic progress is promoted by the engagement in learning tasks, the persistence in spite of difficulties and the development of reflective working habits [17]. All these learning behaviors (LB) are observable patterns of behaviors exhibited by students as they respond to learning situations and react to academic tasks in educational settings. LB are focused on readiness to learn and they increase substantially the information afforded by measures of general intelligence in explaining the variability of students’ academic achievement, social adjustment, and susceptibility to learning disabilities [18,19,20].

Students exhibiting adaptive learning behaviors participate and dedicate efforts to activities, they collaborate in class and show positive attitudes toward learning. Evidence from research in children with ASD indicates that they show a unique constellation of characteristics that represent challenges to learning. For example, children with ASD have difficulties to engage with or respond to their environment [21], they show reduced information seeking when learning new tasks [22] and their engagement overall is low, spending limited time participating in the classroom activities productively and independently [23]. Moreover, approximately 40% of children with ASD present behaviors characteristic of ADHD [24], which negatively affect cognitive, social, and behavioral aspects of child’s functioning and they present greater difficulties than children with ASD without ADHD symptoms, such as more response inhibition challenges [25,26,27]. Attention-related behaviors such as impulsivity, hyperactivity and poor concentration have been associated with impaired academic and social functioning [28], but it has hardly been investigated whether the ADHD symptoms serve as a risk factor for additional learning problems in autism. The only study where this topic has been investigated suggests that HFA children with higher symptoms of ADHD may be at greater risk for writing difficulties than children with HFA with lower symptoms of ADHD. In particular these problems were focused in the theme development and text organization [29]. Furthermore, several studies have showed that disruptive behavior is a major barrier to the acquisition of educational goals in children with autism [30], causing a delay in the onset of the task and decreasing the number of tasks completed, even though the tasks are within their academic capability [31].

Children with ASD and ADHD symptoms also show a weak profile on EF [26] a wide range of higher mental processes that are necessary to guide goal-directed behavior. Conceptually, EF has a strong influence on the academic performance of children with TD and children with neurodevelopmental disorders other than ASD [32,33,34,35], because it may aid concentration and deployment of skills to achieve the learning goals. The association between EF skills and achievement remains stable over time for different age groups, different subcomponents (inhibition, attention control, attention shifting, and working memory), and different measurement types (naturalistic vs. laboratory based) [36]. EF contribute to participation of children diagnosed with high functioning ASD in school activities over and above the contribution of sensory processing [37,38]. More specifically working memory, inhibition and attention shifting have shown a positive relationship with better mathematics performance, reading and language [39,40,41]. Furthermore, there are evidences that show that metacognitive monitoring ability is significantly disminished among children with ASD [42]. Consequently, in general terms, data strongly indicate that EF is one of the regulatory processes that contribute to educational success among children with autism.

The deficits in executive functions (EF) showed by children with ASD that in addition present ADHD are more remarkable even than those of children with ASD, mainly in verbal working memory tasks [43]. The findings about EF behavior ratings in natural contexts show strong deficits too that are more consistent than laboratory-based findings. Therefore, that group experiences a “double hit”, sharing deficits with both “pure” groups, ASD and ADHD [44].

In summary, literature shows that children with ASD have problems with school adjustment and academic performance, possibly related to social and communication difficulties and rigid thinking and behavior. Previous findings also point out the ADHD symptoms [25,26,27] and EF deficits [38,39,40,41] as additional reasons for learning problems in children with ASD. The role of LB, which are actions easily observed by the teachers referred to how children learn rather than how well, has not been investigated. However, the information related to learning behaviors could have important implications to improve readiness to learn of children with ASD. Since these are modifiable factors, knowing the pattern of abilities and impairments will enable the adaptation of teaching strategies and interventions, moving the focus to the ones that promote them from early ages.

The goal of the present study was to examine LB in children with ASD and the relationships between ADHD symptoms, LB, and EF. By analyzing the relationship between these three variables in a mediation frame-work we intent to extend the previous findings on the negative influence of the symptoms of inattention and hyperactivity/impulsivity in the development of children with ASD. The focus is to understand whether there is a link between LB and ADHD symptoms in children with ASD, by assessing whether this relationship is assembled through the EF.

The following two questions were asked in this empirical study: 1) Does the level of development of LB and the symptoms of ADHD differ between children with ASD and children with TD? The hypothesis is that the children with ASD will have more ADHD symptomatology and lower scores than TD children on all the LB: Motivation, Attitude toward learning, Persistence on the task, Strategies/Flexibility; and 2) Are ADHD symptoms, the EF and the LB correlated in children with ASD, with the EF in a mediating role between the ADHD symptoms and LB? The prediction is that LB, ADHD symptoms and EF will maintain association with each other. Moreover it is supposed that both EF components, behavioral regulation and metacognitive components, will mediate in the relationship between ADHD symptoms and all the LB.

## Materials and methods

### Participants

Participants in the present study were 89 children with ages between 7 and 11 years old, enrolled in schools in the Valencian Community. They all had an intellectual capacity (IQ) equal to or above an average score of 70 measured by the K-BIT which includes verbal and non-verbal measures [45], and they were distributed in two groups: a group of 52 children with autism spectrum disorder and a group of 37 children with typical development, matched on age and IQ. Of them, 79.7% were boys, and 20.3% were girls. Table 1 shows the sociodemographic characteristics of the sample.

**Table 1:** Sociodemographic characteristics of the sample.

	TD (n = 37)	ASD (n = 52)	F_1,87_	χ^2^	*p*	η^2^_P_ */ Cramer's V*
	M (SD)	M (SD)				
Age	8.54 (1.26)	8.59 (1.38)	0.02	-	.880	.00
IQ	102.11 (8.91)	101.42(12.65)	0.08	-	.778	.00
Number of Siblings	1.19 (.85)	0.84 (.65)	4.76	-	.032*	.05
Sex (boys) N (%)	23 (62.1)	48 (92.3)	-	12.17	.000*	.37
Medication N (%)	0 (0.00)	17 (32.7)	-	14.95	.000*	.41
Repeated courses N (%)	0 (0.00)	3 (5.76)	-	2.20	.137	.15
Educational Supp N (%)	0 (0.00)	52 (100.0)	-	81.18	.000*	.95
ADI-R A	-	13.49 (2.94)				
ADI-R B	-	8.91 (2.55)				
ADI-R C	-	4.70 (2.03)				
SCQ	3.13 (2.70)	22.51 (7.01)	254.5		.000*	.74
Father’s education	3.08 (1.27)	2.62 (1.39)	2.47	-	.119	.03
Mother’s education	3.44 (1.05)	3.02 (1.30)	2.59	-	.111	.03

*Note*. Father’s education and mother’s education: mean scores (0 = Primary education, 1 = Secondary Education, 2 = High School, 3 = University Degree), Educational Supp = Educational Support; ADI-R: Autism diagnostic interview-revised; ADI-R A: Qualitative alterations in the reciprocal social interaction; ADI-R B: Qualitative alterations in communication; ADI-R C: Restrictive and stereotyped behaviors; SCQ: Social communication questionnaire

*p < .05

The participants with ASD had received a clinical diagnosis in centers specialized in the assessment and treatment of autism and other neurodevelopmental disorders in the Valencian Community (Psychiatry and Neuropediatric units of hospitals and medical centers). To confirm the diagnosis, cut-off scores were used that are recommended on the Social Communication Questionnaire (SCQ), (values ≥ 15) [46] and on the Autism diagnostic interview-revised with diagnostic algorithm (ADI-R), ADI-R A (Qualitative alterations in the reciprocal social interaction) values ≥ 10; ADI-R B (Qualitative alterations in communication) values ≥ 8 and ADI-R C (Restrictive and stereotyped behaviors) values ≥ 3 [47]. The ADI-R was administered by a member of the research team. All of the participants with ASD spent the majority of their time in ordinary classrooms in elementary schools and they received educational support. Specifically, they were in therapeutic support rooms five hours a week in their school. Moreover, 32.7% of the children with ASD were taking medication (mostly antipsychotic and to a lesser extent psychostimulants) for behavioral problems and symptoms of irritability; and 36% of the children with ASD had emotional and behavioral problems assessed by Strengths and Difficulties Questionnaire (SDQ) [48].

The children with TD were enrolled in the same schools as the children with ASD. They had no history of psychopathology or referral to children’s mental health units (USMI), according to information from the school records. The exclusion criteria for all the participants in this study were rated through an extensive prior anamnesis performed with the families. They included neurological or genetic diseases, brain lesions, visual, auditory, or motor sensorial deficits, and an intelligence coefficient below 80.

### Measures

#### Executive functioning- behavior rating inventory of executive function (BRIEF) [49]

The teacher version of the BRIEF was administered. It consists of 86 items that assess the child’s EF through the observation of his/her behavior in the school context. The items are scored on a Likert-type scale with three levels (never, sometimes, often), and they are grouped in 8 scales that make up two general indices. The behavioral regulation index (BRI) assesses the child’s capacity to change his/her affective state and modulate his/her emotions and behavior by using appropriate self-control. It includes the subscales of inhibition, shift and emotional control. The metacognitive index (MI) includes the subscales of initiative, working memory, planning/organization, organization of materials, and monitoring. This index reflects the child’s ability to manage tasks cognitively and supervise their performance.

The direct scores can be transformed into T scores, with higher scores indicating worse executive functioning. The test-retest reliability of the teacher version of the BRIEF ranged between 0.86 and 0.92 on the Spanish adaptation [50]. These values are similar to those obtained on the original version of the test [49]. In our sample, the internal consistency coefficient is high for the total score (α = 0.98), for the two indices (α = 0.96–0.97), and for the subscales (α = 0.78–0.94).

#### Learning behaviors scale (LBS; McDermott et al., 2001) [51]

The instrument used was the LBS, a 29-item, standardized questionnaire for teachers. The purpose of the test is to assess behaviors related to the student’s effective learning in the past two months. The items on the questionnaire, positive and negative, are rated on a Likert-type scale with 3 response options (0 = “Does not apply”, 1 = “Sometimes apply”, 2 = “Most often apply”). The LBS offers 4 dimensions and a total score: Motivation/Competence, which includes behaviors related to the anticipation of success; Attitude toward learning, which measures the willingness to be involved in learning activities; Persistence/attention, which rates persistence on the task and concentration until finishing it; and Learning strategy/flexibility, which evaluates the general way of approaching tasks. High scores indicate good learning behavior. The direct scores are transformed into normalized T scores (mean = 50, standard deviation = 10), based on the standardization of a representative sample of 1,500 students with ages between 5 and 17 years. The internal consistency coefficients are high for the total score (.89, .92) and for the subscales (.70, .87) [52]. In our sample, the internal consistency coefficient is high for both the total score (α = 0.93) and the subscales (α = 0.76–0.86).

#### ADHD Symptoms (DSM-5; American Psychiatric Association, 2013) [1]

An 18-item rating scale of behaviors was used in an interview with the parents and teachers. Nine items describe inattention behaviors, and the other nine describe hyperactivity/impulsivity behaviors, as described in the DSM-5 criteria for ADHD[1]. The items receive scores from 0 (never or rarely) to 3 (quite often). On both versions of the scale, for parents and for teachers, alpha coefficient values were high (0.81–0.90). The sum of all the items was used in the analyses as a dimensional measure of ADHD symptomatology.

### Procedure

The present study was part of more extensive research designed to perform an in-depth examination of different components of cognitive and social processing in children with ASD, ADHD, and typical development. The study was approved by the Ethics Committee of the University of Valencia (Declaration of Helsinki in the Convention of the Council of Europe, 1964). In addition, authorization was given by the Board of Education of the Valencian Government to access the schools and locate the participants. The evaluation was performed in the schools where the children were enrolled. Informed oral consent was also obtained from the children, along with written consent from the parents of all the participants, after being informed of the objectives of the study.

The evaluation was carried out in rooms prepared by the schools that met the optimal conditions for the psycho-educational assessment. The intelligence test was administered individually to all the children by an expert examiner. The parents and teachers provided information about sociodemographic developmental data and the attention and hyperactivity/impulsivity behaviors of their children. The teachers-tutors provided ratings of the EF and learning behaviors in class.

### Data analyses

The statistical analyses were performed with the statistical program for the Social Sciences (SPSS), version 23. Before using the parametric tests, histograms and Kolmogorov-Smirnov tests were conducted to check the normality of the distribution of the variables. To compare the learning behaviors, a multivariate analysis of covariance (MANCOVA) was performed between the TD and ASD groups, using sex as covariate and the subsequently the ADHD score was employed as an additional covariate. The effect size was calculated to test the strength of the association. Table 2 presents group differences and partial eta squared effect sizes, which can be interpreted as small = .01, medium = .06 and large = .14 [53]. In addition, partial correlation analyses were conducted with the ASD group between the different learning behavior variables, the main executive functioning indices (BRI and MI), and the dimensional score for the ADHD symptoms according to the DSM-5, using sex as covariate. Finally, multiple mediation analyses were performed in ASD group to study the mediation effect of the executive functioning indices in the relationship between the ADHD symptoms from the DSM-5 and the different learning behaviors.

**Table 2:** Comparisons of learning behaviors and ADHD symptoms in ASD and TD groups.

	TD (n = 37)	ASD (n = 52)	F_1,85_	*p*	η^2^_P_
	M (SD)	M (SD)			
Motivation	53.11 (9.75)	39.53 (11.11)	31.52	.000*	.26
Attitude	50.57 (7.87)	39.85 (7.31)	29.26	.000*	.25
Persistence	52.43 (8.41)	40.89 (7.81)	32.15	.000*	.27
Strategy	50.43 (8.13)	29.67 (15.31)	43.03	.000*	.33
Total LBS	52.05 (9.02)	35.58 (13.47)	32.50	.000*	.27
ADHD S	12.64 (8.26)	49.31 (16.38)	129.4	.000*	.60

*Note*. M: Mean, SD: (Standard deviation); ADHD S: ADHD symptoms

*p = .008 (Bonferroni correction)

In the mediation analyses, the bootstrap procedure with 10,000 repetitions was used to verify the mediator effect of the aforementioned variables with a confidence interval of 95%. When the indirect effect of the mediators is significantly different from zero, this indirect effect is considered significant.

Following Baron and Kenny [54], in order for the model to be acceptable, four assumptions must be met: a) the predictor variable must be related to the dependent variable; b) the predictor variable must be related to the mediator; and c) the mediator variable must be related to the dependent variable after controlling the effect of the predictor variable. The final condition is that the effect of the predictor variable on the dependent variable, controlling the effect of the mediator variable, must not be statistically significant. In the present study, due to the sample size, we followed the recommendations of MacKinnon, Lockwood and Williams [55], designed specifically to obtain reliable and valid conclusions in studies where the sample samples are not large [56].

## Results

### Differences in learning behaviors and ADHD symptoms of children diagnosed with ASD and TD

The MANCOVA carried out to evaluate the main effect of group on the learning behavior indicators, with the sex covariate, was statistically significant [Wilk‘s Lambda (Λ) = .37, F_(6,80)_ = 22.45, *p* < .001, η^2^p = .62]. The confirmation ANCOVAs showed statistical significance on all the variables (*p* < .001): Motivation toward competence, attitudes toward learning, persistence on the task and strategy/flexibility. ASD group showed lower scores than TD group (see Table 2).

Additionally, we introduce the ADHD symptoms as a covariate in the MANCOVA in order to see the generic effect upon the group differences in the LBS. In this case the differences between both groups became not significant (Motivation *p =* .*147*, η^2^_P_ = .025; Attitude *p =* .*267*, η^2^_P_ = .014; Persistence *p =* .*457*, η^2^_P_ = .007; Strategy *p =* .*266*, η^2^_P_ = .015; Total LBS *p =* .*400*, η^2^_P_ = .008).

These results were expanded by mediation analysis in order to identify more finely the relationship between ADHD symptoms, EF and LBS in the ASD group.

### Mediation effect of executive functioning on the learning behaviors

Previously to mediation analysis, Table 3 shows the correlations among the study variables in the ASD group. Statistically significant values were found in the theoretically expected direction.

**Table 3:** Partial correlations between executive functions, ADHD symptoms and learning behaviors in ASD group.

			1	2	3	4	5	6	7
ASD	1.S. ADHD		-						
	2.BRI		.42*	-					
	3.MI		.58**	.53**	-				
	4.Motivation		-.24	-.46*	-.59**	-			
	5.Attitude		-.40*	-.48**	-.61**	.71**	-		
	6.Persistence		-.56**	-.49**	-.75**	.60**	.72**	-	
	7.Strategy		-.47*	-.68**	-.45*	.37*	.57**	.56**	-

*Note*. Controlling for gender; BRI: Behavioral regulation index; MI: Metacognitive Index; ADHD S.: ADHD symptoms

*p < .05

** *p* < .01

In the ASD group, the ADHD symptoms were significantly correlated with the BRI (*p* = .002), the MI (*p* < .001), attitude (*p* = .005), persistence (*p* < .001), and strategy (*p* = .001). Likewise, significant associations were observed between the BRI and the subscales of the LBS, motivation (*p* = .001), attitude (*p* < .001), persistence (*p* < .001), and strategy (*p* < .001), and between the MI and motivation (*p* < .001), attitude (*p* < .001), persistence (*p* < .001), and strategy (*p* = .001).

In sum, the correlation analyses supported the viability of the mediation analyses with the main executive indices (BRI and MI) as mediator variables in the ASD group.

Mediation analyses were performed using the PROCESS program of mediation, moderation, and conditional analysis [57], in order to examine the mediation of the BRI and MI executive indices in the relationship between the ADHD symptoms and the learning behaviors of motivation, attitude, persistence, and strategy, in the children with ASD. For this purpose, three multiple mediation analyses were conducted in ASD group, including sex as covariate. The independent variable used was the dimensional score for ADHD symptoms from the DSM-5, the T-scores for the subscales of LBS that presented a significant correlation with the ADHD symptoms in ASD group were dependent variables, and the BRI and MI were partial mediator variables.

**Fig 1:**
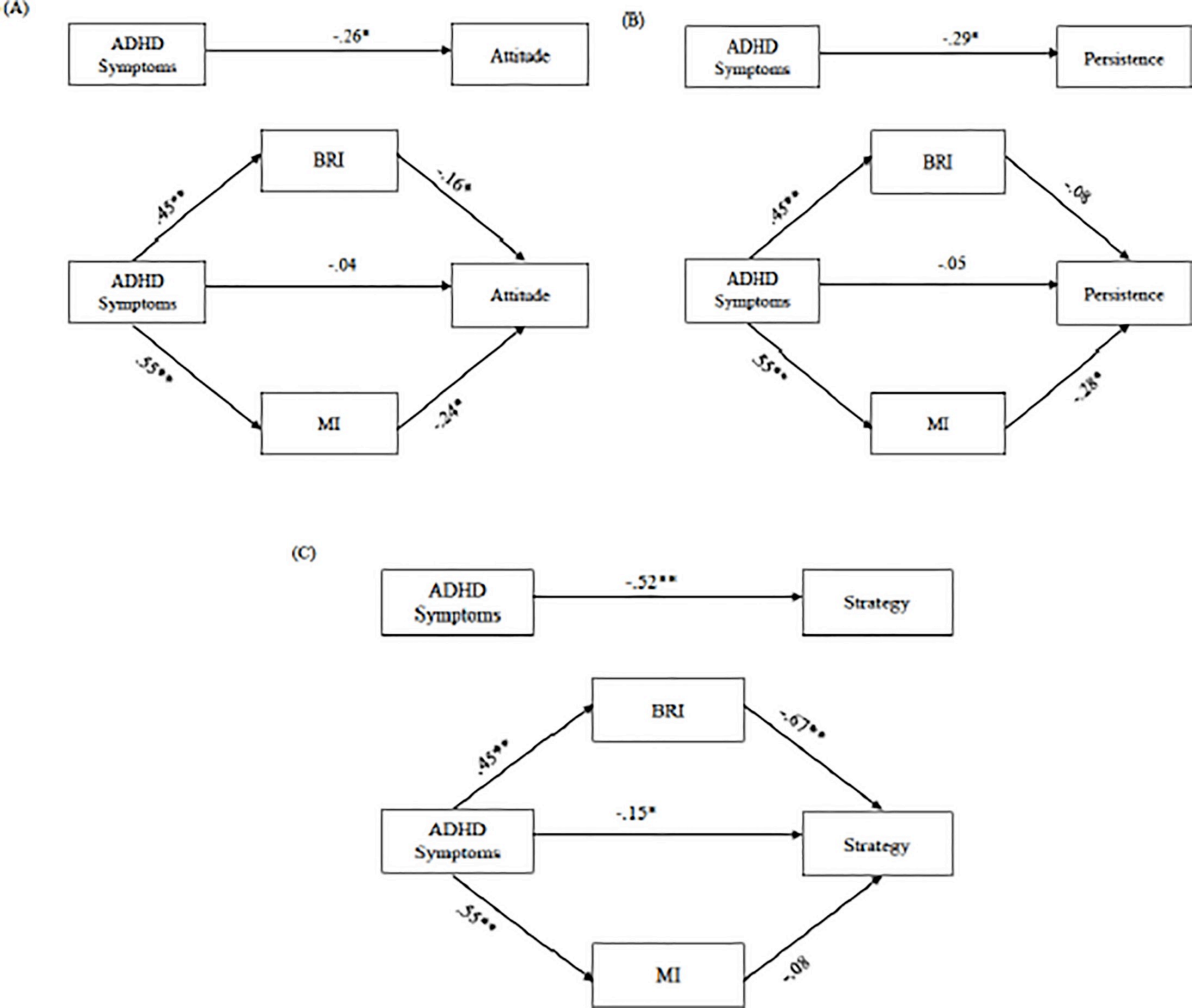
*p < .05, ** *p* < .01*Note*. Multiple mediation analyses in ASD group of direct effect of the ADHD symptoms on the attitude controlling for the two executive mediators (A), on the persistence controlling for the two executive mediators (B), on the strategy controlling for the two executive mediators (C).

Fig 1(A) shows in the ASD sample that when the direct effect of the ADHD symptoms on the attitude toward learning was evaluated, controlling for the two executive mediators, the result was not statistically significant [-.112, .141]. In this case, the indirect effect obtained with the mediator MI, using the *bootstrapping* procedure for a sample of 10,000 and a 95% confidence interval (CI), was statistically significant, CI [.004, .211]. Similar results were observed when the direct effect of the ADHD symptoms on persistence in learning was evaluated (B), controlling for the BRI and the MI. Statistically significant indirect effects were found with the mediator MI, CI [.003, .225], and with the two mediators, BRI and MI, together, CI [.026, .232]. By contrast, Fig 1(C) shows that there were statistically significant indirect effects of mediation with the BRI mediator, CI [-.450, -.128], and with the two mediators, BRI and MI, together, CI [-.471, -.059]. In this case, the direct effect of the ADHD symptoms on the learning strategy/flexibility, controlling for the two executive mediators, was statistically significant (*p* = .046), so that the mediation was partial. The analysis of the covariate used indicated that the sex did not have a significant impact (β = 2.09, *p* = .65).

## Discussion

Children with ASD are increasingly being included in ordinary classrooms in Spain, following the tendency of other European countries and the United States. Consequently, schools face the challenge of providing appropriate services to respond to the needs of students with this neurodevelopmental disorder. Given the many challenges faced by children with autism in the school setting, it is important to understand the factors that underlie to their classroom school functioning. The present study represents an attempt to collaborate in advancing in this direction, completing the contribution of perspectives focused on school grades or standardized test scores with the analysis of LB in children with ASD.

Confirming the first hypothesis, teachers evaluated children with ASD as having a lower level that the children with TD in all learning behaviors which allow students to get educational goals in the classroom. According to their teachers, the children with ASD showed typical behaviors of insufficient motivation, such as less willingness to complete tasks and little perseverance, giving up when encountering difficulties without making an effort to overcome them. Likewise, the teachers reported that the children with ASD had a more negative attitude toward learning, which was shown, for example, in their resistance to taking on a new task. Finally, but not less important, the children with ASD adopted peculiar and inflexible procedures in performing tasks, which materialized as difficulties in shifting from one task to another, modifying a procedure according to a new objective, or trying to find new solutions to problems. On the whole, this learning style is framed in the constellation of restrictive behaviors and interests that are part of the definition of the disorder in the DSM- 5 [1].

Importantly, our findings extend previous work supporting that children with autism have impairments in significant components of school success, including behavioral school engagement, school liking, emotional school engagement [58, 21, 23, 22]. They also show coincidences with other studies comparing LB in children with TD and children with neurodevelopmental disorders, such as ADHD [59,60]. Taken together these results reveal the importance of examining the possible impairments of students with ASD in a set of skills that act as a foundation for learning in the classroom, - motivation, attitude toward learning, persistence and flexibility-, which according teachers may be considered even more important than academic skills [61].

Likewise, the comparison of ADHD symptoms in the group of children with ASD and the group with TD yielded the expected results, coinciding with empirical studies that show the presence of ADHD symptoms in children with ASD [24,62,63]. The cognitive, social, and behavioral implications of this association are great. To date, studies of individuals with ASD and ADHD symptoms suggest that there are greater cognitive, social, and behavioral impairments in children with both disorders than in those with ASD alone [25,26,27]. In any case disruptive behaviors are additional risk factors for learning problems in ASD [30,31].

In order to advance the understanding of the manifestations of ADHD symptoms and EF in LB of students with ASD, the second question of this study addressed the analysis of the relationships among all these variables. On the one hand, the results supported significant and negative associations between ADHD symptoms and LB in children with ASD. Only the relationship between motivation and ADHD symptoms did not achieve statistical significance, although it was close, which suggests that curiosity about learning activities does not have a strong relationship with inattention or hyperactivity/ impulsivity behaviors in students with ASD. On the other hand, consistent with the literature, a significant relationship between ADHD symptoms and EF [26] was found. In addition, the association between EF and LB was confirmed, suggesting the importance of metacognition and behavioral regulation behaviors in academic enablers in children with ASD [40,41,42].

The idea that EF deficits could be related to the LB of children with ASD is justified when considering the contextual demands of the school setting. Therefore, it is interesting to explore what specific facets of EF, behavioral regulation, and metacognition, were mediating the relationships between ADHD symptoms and different LB. A strong point of this empirical study, which to our knowledge has not been addressed until now, consisted of the analysis of the possible mediator role of EF in the LB of children with ASD, that is, in the attitudes and behaviors that allow them to participate in and benefit from instruction in class. The results of the multiple mediation analysis carried out were coherent with the prediction. Specifically, the metacognitive processes, that is, the amalgam of working memory, planning, initiative, organization of materials, and supervision skills, mediated the relationship between the ADHD symptoms and the attitude toward learning. Along the same lines, the metacognitive processes were also a significant mediator of persistence on the task. In other words, metacognitive executive functions explained the attitudes toward learning and the persistence on the task, above and beyond the dimensions of inattention/disorganization and hyperactivity/impulsivity. In addition, the results of the multiple mediation analysis showed the importance of the behavioral regulation index. However, there was only partial mediation between ADHD symptoms and the strategy/flexibility learning. Therefore, success on non-routine problem-solving tasks that require flexible thinking and the generation of new problem-solving strategies depends, at least partly, on skills such as inhibition, shifting, and emotional control. Precisely, as has been noted, more than 50% of children and adolescents with ASD have clinically significant cognitive flexibility deficits in the BRIEF [64]. These impairments may partly are provoking difficulties in the use of the strategy/flexibility learning.

In summary, the findings showed significant mediation effects of the BRI index and MI index of EF, suggesting that both are part of the path (mechanisms) through which ADHD symptoms affect LB in children with ASD. In other words, these impairments are not only a function of diagnosis of ASD but are also a consequence of the combination of the ASD and ADHD characteristics. There seems to be a unique contribution of specific aspects of EF in predicting different learning behaviors of children with ASD, with metacognition being more related to attitudes toward learning and persistence to task, and behavioral regulation being more related to strategy/flexibility. Thus, the appropriate development of the metacognitive processes of the EF may promote the tendency towards being proactive, as well as abilities to stay on task, ignore distractions, and persist despite the difficulties. On the other hand, the executive functioning behaviors of behavioral regulation might play an essential role in the child's capacity to select coping strategies, and to develop strategies effectively to a specific learning situation. In fact, behavioral regulation has previously been revealed as a salient index of self-regulation for both emotional and behavioral school engagement [58].

### Limitations

Our study has some limitations that must be mentioned. The measures of EF and the learning behaviors used in the current study relied primarily on teacher reports, that may have been negatively influenced by knowing the ASD diagnosis. However, we trust that the knowledge of the difficulties that ASD implies and its consideration as a “special educational needs” in the education system might have avoided to a large extent the possible negative bias in the teacher’s assessment.

Although the information was provided by informants who are very familiar with the behavior of the individuals in relevant contexts for academic and social learning, it would have been desirable to complement this information with observations made in the classroom and/or home. Results may have been different if observational or performance measures related to a learning behavior and executive functions were utilized. In fact, the agreement between neuropsychological tasks of EF and parents and teacher’s ratings using the BRIEF shows moderate correlation values between these two different assessments procedures [65,66]. As the review by Toplak et al. [67] underlined, performance-based and ratings measures of EF capture different level of analysis or underlying processes, so they should not be used interchangeably as parallel measures in clinical or educational assessments. They are complementary measures. Daily life situations ratings inform about the individual behaviour in order to address multiple cognitive and social demands. They have ecological validity and they could contribute to a broader understanding of EF than the performance in neuropsychological tests in laboratory settings. But, in any case, real-world EF impairments observed by teachers would not reduce the importance of neuropsychological tasks because both measures may reflect the child’s performance in various environmental contexts. Certainly, the use of a battery of neuropsychological tests in our study might have allowed a more complete and detailed examination of the relationship between EF skills and learning behaviors.

Additional measures of student academic performance were also not included and future research is needed to document the relations between the EF, ADHD symptoms, learning behaviors and additional measures of classroom academic performance. The small sample size, particularly of the TD group, and the major sampling bias towards males in the ASD sample represent a limitation of the study. The results cannot be generalized to girls with ASD either, given the low number of female participants. This limitation is not unique to our study, however, it must be a goal for future research on ASD as findings so far are scarce, suggesting a relative weakness for females compared to males diagnosed with ASD on executive function and daily living skills [68]. These differences between females and males with ASD on EF may affect the results of the mediation models. Moreover, the results are only applicable to children with an IQ equal to or above 80, limiting their generalization to a broader population of children with ASD.

In addition, the transactional processes that take place in the classroom are difficult to rate and are not captured in our study. For example, it would also have been important to explore other factors associated with a positive atmosphere in the classroom, such as the teacher’s degree of closeness [69] and the beliefs teachers hold regarding their capability to bring about desired instructional outcomes for students with ASD [70]. It would have been relevant to analyze if there are differences on learning behaviors depending on variables of family environment. In fact, a recent study shows [71] that there are associations between parental disciplinary style and child self regulation in children with ASD, which is an important requirement for social functioning in general and for success in educational settings.

### Conclusion and future directions

Despite these limitations, the current study makes an important contribution to our knowledge about implications of executive functions in learning behaviors of children with ASD, underlining the importance for designing interventions that fit their special educational needs. Efforts to increase EF in students with ASD in educational settings could be fundamental to promote LB such as motivation / competence, attitudes towards learning, persistence on tasks and strategy flexibility. There are some positive data in this direction. For example, a recent work demonstrated that the application of the Cogmed Working Memory Training improved the attention, impulsivity, emotional reactivity and academic achievement in children with ASD and comorbid ADHD [72].

However, interventions in the common environments of children with ASD and ADHD have more guarantees of effectiveness and generalization of skills than those implemented in a clinical context. As experts point out, the problem of ADHD is not a problem of lack of knowledge but rather of the application of what is known in the context and at the right moment, due to a EF deficit [73]. In the school context, Kenworthy et al. [74] developed an intervention based on EF to teach cognitive/physical flexibility, goal setting, and planning, through experiments, discussions of scenarios, and the use of self-regulation scripts. Kenworthy’s program, which gives an essential role to the school, is not an isolated effort. It connects with new approaches and intervention objectives that assume the conceptualization of ASD as a form of social learning disability that affects learning [75]. From these perspectives, the success of ASD interventions depends to a large extent on their ability to create optimal learning environments to achieve social and personal well-being. It would be advisable to promote early interventions to improve learning behaviors of children with HFA before they transit to a formal learning environment. The program "Tools of the Mind" [76] for preschool children is based on the socio-constructivist theory of development and aims to promote three executive functions (working memory, inhibitory control and flexibility) through mature and intentional play. The Tools of the Mind curriculum is found to improve the classroom experiences, social development and academic success of at-risk young children [77], thereby supporting the possible effectiveness of its application with preschoolers with ASD. Early intervention is the best procedure to improve a child's long-term outcomes and reduce lifetime costs to the individual, family and society [78].

## Supporting information

S1 File Data. (XLS)

## References

[1] American Psychiatric Association Diagnostic and Statistical Manual of Mental Disorders (DSM-5) 5th ed. Arlington, VA: American Psychiatric Publishing; 2013.

[2] Tick B, Bolton P, Happé F, Rutter M, Rijsdijk. Heritability of autism spectrum disorders: a meta-analysis of twin studies. J Child Psychol Psychiatry. 2016; 57 (5): 585–595. 10.1111/jcpp.12499 26709141 10.1111/jcpp.12499 PMC4996332

[3] Bieleninik Ł, Posserud M-B, Geretsegger M, Thompson G, Elefant C, Gold C. Tracing the temporal stability of autism spectrum diagnosis and severity as measured by the Autism Diagnostic Observation Schedule: A systematic review and meta-analysis. PLoS One. 2017; 12(9): e0183160 10.1371/journal.pone.0183160 28934215 PMC5608197

[4] U.S. Department of Health and Human Services. Prevalence of autism spectrum disorder among children aged 8 years—Autism and developmental disabilities monitoring network, 11 sites, United States, 2010. Morbidity and Mortality Weekly Report. Surveillance Summaries (Washington, DC: 2002). 2014; 63: 1–21. Retrieved from https://doi.org/24670961 24670961

[5] Loomes R., Hull L., & Mandy W. P. L. What is the male-to-female ratio in autism spectrum disorder? A systematic review and meta-analysis. J Am Acad Child Adolesc Psychiatry 2017;56: 466–474. 10.1016/j.jaac.2017.03.013 28545751 10.1016/j.jaac.2017.03.013

[6] Baio J, Wiggins L, Christensen DL, Maenner MJ, Daniels J, Warrens Z, et al Prevalence of autism spectrum disorder among children aged 8 years. Autism and developmental disabilities monitoring network, 11 Sites, United States, 2014. Centers for disease control and prevention morbidity and mortality weekly report. Surveill Summ. 2018; 67 (6): 1–23. doi: 10.15585/mmwr.ss6706a1 29701730 10.15585/mmwr.ss6706a1 PMC5919599

[7] Christensen DL, Baio J, Braun KV. Prevalence and characteristics of autism spectrum disorder among children aged 8 years-Autism and Developmental Disabilities Monitoring Network, 11 Sites, United States, 2012. MMWR Surveill Summ. 2016; 65: 1–23. 10.15585/mmwr.ss6503a1 PMC7909709 27031587

[8] Howlin P, Goode S, Hutton J, Rutter M. Adult outcome for children with autism. J Child Psychol Psychiatry. 2004; 45: 212–229. 14982237 10.1111/j.1469-7610.2004.00215.x

[9] Keen D, Webster A and Ridley G. How well are children with autism spectrum disorder doing academically at school? An overview of the literature. Autism. 2016; 20 (3): 276–294. 10.1177/1362361315580962 25948598 10.1177/1362361315580962

[10] Kimhi Y (2013) Cognitive strengths and weaknesses In Bauminger-Zviely N.(Ed), Social and academic abilities in children with high functioning autism spectrum disorder (pp110–130) New York: The Guilford Press.

[11] Oswald TM, Beck JS, Iosif AM, McCauley JB, Gilhooly LJ, Matter JC, et al Clinical and cognitive characteristic associated with mathematics problem solving in adolescents with autism spectrum disorders. Autism Res. 2016; 9 (4): 480–490. 10.1002/aur.1524 26418313 10.1002/aur.1524 PMC6910857

[12] Troyb E, Orinstein A, Tyson K, Helt M, Eigsti IM, Stevens M, et al Academic abilities in children and adolescents with a history of Autism Spectrum Disorders who have achieved optimal outcomes. Autism. 2014; 18(3): 233–243. 10.1177/1362361312473519 24096312 10.1177/1362361312473519 PMC4481875

[13] Tirado MJ, Saldaña D. Readers with autism can produce inferences, but they cannot answer inferential questions. J Autism Dev Disord. 2016; 46 (3):1025–1037. 10.1007/s10803-015-2648-6 26547920 10.1007/s10803-015-2648-6

[14] Mayes SD, Calhoun S. Ability profiles in children with autism. Influence of age and IQ. Autism.2003; 7 (1): 65–80. 10.1177/1362361303007001006 12638765 10.1177/1362361303007001006

[15] Jones C, Happe F, Golden H, Marsden AJ, Tregay J, Simonoff E, et al Reading and arithmetic in adolescents with autism spectrum disorders. Peak and dips in attainment. Neuropsychol. 2009; 23(6): 718–728. 10.1037/a0016360 19899830 10.1037/a0016360

[16] DiPerna JC, Elliott SN. Promoting academic enablers to improve student achievement: Introduction to a miniseries. Sch Psychol Rev. 2002; 31(3): 293–297. Retrieved from https://search.proquest.com/docview/219653644

[17] Li-Grining CP, Votruba. Drzal E, Maldonado Carreño C, Haas K. Children´s early approaches to learning and academic trajectories through fith grade. Dev Psycho. 2010; 46 1062–1077.doi:10.1037/a0020066 10.1037/a0020066 20822223

[18] McDermott PA, Rikoon SH, Fantuzzo JW. Transition and protective agency of early childhood learning behaviors as portents of later school attendance and adjustment. J Sch Psychol. 2016; 54: 59–75. 10.1016/j.jsp.2015.10.003 26790703 10.1016/j.jsp.2015.10.003

[19] Sasser TR, Bierman KL, Heinrichs B. Executive functioning and school adjustment: the mediational role of pre-kindergarten learning-related behaviors. Early Child Res Q. 2015; 30: 70–79. 10.1016/j.ecresq.2014.09.001 27231409 10.1016/j.ecresq.2014.09.001 PMC4878834

[20] Yen C, Konold TR, McDermott PA. Does learning behavior augment cognitive ability as an indicator of academic achievement? J Sch Psychol. 2004; 42: 157–169.

[21] Olley JG, Reeve CE. Issues of curriculum and classroom structure In Cohen D. J & Volkmar F. R (Eds.), Handbook of autism and pervasive developmental disorders (2nd ed., pp. 484–508). New York: Wiley; 1997.

[22] Young N, Hudry K, Trembath, Vivanti G. Children with autism show reduced information seeking when learning new tasks. Am Intellect Dev Disabil. 2016; 121(1): 65–73. 10.1352/1944-7558-121.1.65 . 26701075 10.1352/1944-7558-121.1.65

[23] Sparapani N, Morgan L, Reinhardt VP, Schatschneider C, Wetherby AM. Evaluation of classroom active engagement in elementary students with autism spectrum disorder. J Autism Dev Disord. 2016; 46 (3): 782–796. 10.1007/s10803-015-2615-2 26433878 10.1007/s10803-015-2615-2

[24] Berenguer C, Miranda A, Pastor G, Roselló R. Comorbilidad del trastorno del espectro autista y el déficit de atención con hiperactividad. Estudio de revisión. Rev Neurol. 2015; 6: S37–43. pmid: 25726822 25726822

[25] Ashwood KL, Tye C, Azadi B, Cartwrights S, Asherson P, Bolton P. Brief Report: Adaptive Functioning in Children with ASD, ADHD and ASD + ADHD. J Autism Dev Disord. 2015; 45 (7): 2235–2242. 10.1007/s10803-014-2352-y 25614019 10.1007/s10803-014-2352-y

[26] Craig F, Margari F, Legrottaglie AR, Palumbi R, de Giambattista C, Margari L. A review of executive function deficits in autism spectrum disorder and attention-deficit/hyperactivity disorder. Neuropsychiatr Dis Treat. 2016; 12: 1191–1202. 10.2147/NDT.S104620 . 27274255 10.2147/NDT.S104620 PMC4869784

[27] Mansour R, Dovi AT, Lane DM, Loveland KA, Pearson DA. ADHD Severity as it relates to comorbid psychiatric symptomatology in children with autism spectrum disorders (ASD). Res Dev Dis. 2017; 60: 52–64. 10.1016/j.ridd.2016.11.009 27889487 10.1016/j.ridd.2016.11.009 PMC5441885

[28] Fleming AP, McMahon RJ. Developmental context and treatment principles for ADHD among college student. Clin Child Fam Psychol Rev. 2012; 15(4): 303–339. 10.1007/s10567-012-0121-z 23053445 10.1007/s10567-012-0121-z

[29] Zajic MC, McIntyre N, Swain-Lerro L, Novotny S, Oswald T, Mundy P. Attention and written expression in school-age, high-functioning children with autism spectrum disorders. Autism. 2016; 22 (3): 245–258. 10.1177/1362361316675121 27940570 10.1177/1362361316675121

[30] Gunter PL, Denny RK, Jack SL, Shores RE, Nelson CM. Aversive stimuli in academic interactions between students with serious emotional disturbance and their teachers. Behav Disord. 1993; 18: 265–274.

[31] Koegel LK, Sing AK, Koegel RL. Improving motivation for academics in children with autism. J Autism Dev Disord. 2010; 40: 1057–1066. 10.1007/s10803-010-0962-6 20221791 10.1007/s10803-010-0962-6 PMC2926912

[32] Barkley RA. Executive functioning and self-regulation Integration, extended phenotype and clinical implications. New York, NY: Guilford Press; 2011.

[33] Engel de Abreu PMJ, Abreu N, Nikaedo CC, Puglisi ML, Tourinho CJ, Miranda MC, et al Executive functioning and reading achievement in school: A study of Brazilian children assessed by their teachers as “poor readers”. Front Psychol. 2014; 5:550 10.3389/fpsyg.2014.00550 24959155 10.3389/fpsyg.2014.00550 PMC4050967

[34] Miranda A, Meliá A, Marco R. Mathematical abilities and executive function in children with attention deficit hyperactivity disorder and learning disabilities in mathematics. Psicothema. 2009; 21 (1): 63–69. 19178858

[35] Sjöwall D, Bohlin G, Rydell A, Thorell LB. Neuropsychological deficits in preschool as predictors of ADHD symptoms and academic achievement in late adolescence. Child Neuropsychol. 2017; 23: 111–128. 10.1080/09297049.2015.1063595 26212755 10.1080/09297049.2015.1063595 PMC5214099

[36] Will E, Fidler DJ, Daunhauer L, Gerlach-McDonald B. Executive function and academic achievement in primary–grade students with Down syndrome. J Intellect Disabil Res. 2017; 61: 181–195. 10.1111/jir.12313 27561217 10.1111/jir.12313

[37] Jacob R, Parkinson J. The potential for school-based intervention that target executive function to improve academic achievement: A review. Rev Education Res. 2015; 85(4): 512–552. 10.3102/0034654314561338

[38] Zingerevich C, LaVesser PD (2009) The contribution of executive functions to participation in school activities of children with high functioning autism spectrum disorder. Res Autism Spec Disord 3: 429–437.

[39] Assouline SG, Foley Nicpon M, Dockery L. Predicting the academic achievement of gifted students with autism spectrum disorder. J Autism Dev Disord. 2012; 42 (9): 1781–1789. 10.1007/s10803-011-1403-x 22105142 10.1007/s10803-011-1403-x

[40] St John TS, Dawson G, Estes A. Brief report: executive function as a predictor of academic achievement in school-aged children with ASD. J Autism Dev Disord.2018; 48(1):276–283. 10.1007/s10803-017-3296-9 28889315 10.1007/s10803-017-3296-9 PMC5762405

[41] May T, Rinehart N, Wilding J, Cornish K. The role of attention in the academic attainment of children with autism spectrum disorder. J Autism Dev Disord. 2013; 43 (9): 2147–2158. 10.1007/s10803-013-1766-2 23378062 10.1007/s10803-013-1766-2

[42] Granger C, Williams DM, Lind SE. Metacognition monitoring and control processes in children with autism spectrum disorder: Diminished judgement of confidence accuracy. Conscious Cogn. 2016; 42:65–74. 10.1016/j.concog.2016.03.003 26985883 10.1016/j.concog.2016.03.003

[43] Takeuchi A., Ogino T., Hanafusa K., Morooka T., Oka M., Yorifuji T., et al Inhibitory function and working memory in attention defcit/hyperactivity disorder and pervasive developmental disorders: Does a continuous cognitive gradient explain ADHD and PDD traits Acta Med Okayama. 2013;67:293–303. doi: 10.18926/AMO/51865 24145729 10.18926/AMO/51865

[44] Dajani DR, Llabre MM, Nebel MB, Mostofsky SH, Uddin LQ. Heterogeneity of executive functions among comorbid neurodevelopmental disorders. Scientific Reports 2016; 6, 36566 10.1038/srep36566 27827406 10.1038/srep36566 PMC5101520

[45] Kaufman AS, Kaufman NI. K-BIT, Test breve de inteligencia de Kauffman [Kaufman brief intelligence test] Madrid: Pearson; 2000.

[46] Rutter M, Bailey A, Lord C. Social communication questionnaire Los Angeles, CA: Western Psychological Services; 2003a.

[47] Rutter M, Le Couteur A, Lord C. ADI-R. Autism diagnostic interview revised Manual. Los Angeles: Western Psychological Services; 2003b.

[48] Goodman R. The strengths and difficulties questionnaire: A research note. J Child Psychol Psychiatry. 1997; 38: 581–586. 10.1111/j.1469-7610.1997.tb01545.x 9255702 10.1111/j.1469-7610.1997.tb01545.x

[49] Gioia GA, Isquith PK, Guy SC, Kenworthy L. Test review behavior rating inventory of executive function. Child Neuropsychol. 2000; 6 (3): 235–238. 0929-7049/00/0603- 10.1076/chin.6.3.235.3152 11419452 10.1076/chin.6.3.235.3152

[50] Maldonado Belmonte MJ. Adaptation of the behavior rating inventory of executive function (BRIEF) to the Spanish population and its usefulness for the diagnosis of attention deficit hyperactivity disorder inattentive and combined subtypes. Doctoral dissertation. Universidad Complutense de Madrid: Spain; 2016.

[51] McDermott PA, Green LF, Francis JM. Learning behaviour scale, Philadelphia, PA: Edumetric and Clinical Service; 2001.

[52] McDermott PA. National scales of differential learning behaviors among American children and adolescents. School Psychol Rev. 1999; 28 (2): 280–291.

[53] Cohen J. Statistical power analysis for the behavioral sciences (1st ed.) New York: Academic Press; 1969.

[54] Baron RM, Kenny DA. The moderator-mediator variable distinction in social psychology research: Conceptual, strategic and statistical considerations. J Person Soc Psychol. 1986; 51: 1173–1182. 10.1037//0022-3514.51.6.1173 3806354

[55] MacKinnon DP, Lockwood CM, Williams J. Confidence limits for the indirect effect: Distribution of the product and resampling methods. Multivariate Behav Res. 2004; 39 (1): 99–128. 10.1207/s15327906mbr3901_4 20157642 10.1207/s15327906mbr3901_4 PMC2821115

[56] Preacher KJ, Hayes AF. SPSS and SAS procedures for estimating indirect effects in simple mediation models. Behavior Research Methods, Instruments & Computers. 2004; 36 (4): 717–731. 10.3758/bf03206553 15641418

[57] Hayes AF. Introduction to mediation, moderation, and conditional process analysis: a regression-based approach New York, NY: The Guilford Press; 2013.

[58] Jahromi LB, Bryce CI, Swanson J. The importance of self-regulation for the school and peer engagement of children with high-functioning autism. Res Autism Spec Disord. 2013; 7: 235–246. 10.1016/j.rasd.2012.08.012

[59] Colomer C, Berenguer C, Roselló B, Baixauli I, Miranda A. The impact of inattention and hyperactivity/impulsivity symptoms and executive function on learning behaviors of children with ADHD. Front Psychol. 2017; 8:540 10.3389/fpsyg.2017.00540 . 28446885 10.3389/fpsyg.2017.00540 PMC5388736

[60] Demaray MK, Jenkins LN. Relations among academic enablers and academic achievement in children with and without high levels of parent related symptoms of inattention, impulsivity, and hyperactivity. Psychol School. 2011; 48: 573–586. 10.1002/pits.21830

[61] Schaefer B, Shur KF, Macri-Summers M, MacDonald S. Preschool Children's Learning Behaviors, Concept Attainment, Social Skills, and Problem Behaviors: Validity Evidence for Preschool Learning Behaviors Scale Scores. J Psychoed Assessm. 2004; 22: 5–32.

[62] Leitner Y. The co-occurrence of autism and attention deficit hyperactivity disorder in children–what do we know? Front Hum Neuros. 2014; 8: 268 10.3389/fnhum.2014.00268 10.3389/fnhum.2014.00268 PMC4010758 24808851

[63] Lyall K, Schweitzer J B, Schmidt R J, Hertz-Piciotto I, Salomon M. Inattention and hyperactivity in association with autism spectrum disorders in the CHARGE study. Res Autism Spectr Disord. 2017; 35: 1–12. 10.1016/j.rasd.2016.11.011 29276530 10.1016/j.rasd.2016.11.011 PMC5738931

[64] Van den Bergh S F W M, Scheeren A M, Begeer S, Koot H M, Geurts H M. Age related differences of executive functioning problems in everyday life of children and adolescents in the autism spectrum. J Autism Dev Disord. 2014; 44: 1959–1971. Do1: 10. 1007/s 10803-014-2071-4. 10.1007/s10803-014-2071-4 24562693 10.1007/s10803-014-2071-4

[65] Miranda A, Colomer C, Mercader J, Fernández I, Presentación MJ. Performance-based tests versus behavioral ratings in the assessment of executive functioning in preschoolers: associations with ADHD symptoms and reading achievement. Front Psychol. 2015; 6 10.3389/fpsyg.2015.00545 10.3389/fpsyg.2015.00545 PMC4413519 25972833

[66] Parrish J, Geary E, Jones J, Seth R, Hermann B, Seidenberg M. Executive functioning in childhood epilepsy: parent report and cognitive assessment. Dev Med Child Neurol 2007; 49: 412–416. 10.1111/j.1469-8749.2007.00412.x 17518924 10.1111/j.1469-8749.2007.00412.x

[67] Toplak M, West R, Stanovich KE. Practitioner review: do performance-based measures and rating of executive function asess the same construct? J Child Psychol Psychiatry 2013; 54: 131–143. 10.1111/jcpp.12001 23057693 10.1111/jcpp.12001

[68] White EI, Wallace GL, Bascom J, Armour AC, Register-Brown K, Popal HS, et al Sex differences in parent-reported executive functioning and adaptive behavior in children and young adults with autism spectrum disorder. Autism Res. 2017; 10 (10): 1653–1662. 10.1002/aur.1811 10.1002/aur.1811 PMC5721669 28568910

[69] García Bacete FJ, Ferrá P, Monjas MI, Marande G.Teacher-Students Relationships in First and Second Grade Classrooms. Adaptation of the Questionnaire on Teacher Interaction-Early Primary (QTI-EP). Psicodidact Rev. 2014;19 (1): 211–231. 10.1387/RevPsicodidact.9081

[70] Ruble LA, Usher EL, McGrew JH. Preliminary investigation of the sources of self-efficacy among teachers of students with autism. Focus Autism Other Dev Disab. 2011; 26: 67–74. 10.1177/1088357610397345 10.1177/1088357610397345 21691453 10.1177/1088357610397345 PMC3117587

[71] Ostfeld-Etzion S, Feldman R, Hirschler-Guttenberg Y, Laor N, Golan O. Self-regulated compliance in preschoolers with autism spectrum disorder: The role of temperament and parental disciplinary style. Autism. 2016; 20 (7): 668–878. 10.1177/1362361315615467 . 26685197 10.1177/1362361315615467

[72] Weckstein SM, Weckstein EJ, Parker CD, Westerman MW. A retrospective chart analysis with follow-up of cogmed working memory training in children and adolescents with autism spectrum disorder. Med Sci Monit Basic Res. 2017; 23: 336–343. doi: 10.12659/MSMBR.904930 . 29033450 10.12659/MSMBR.904930 PMC5656100

[73] Barkley R A. ADHD and the nature of self-control New York: The Guilford Press; 1997.

[74] Kenworthy L, Anthony LG, Naiman DQ, Cannon L, Wills MC, Luong-Tran C, et al Randomized Controlled Effectiveness Trial of Executive Function Invention for Children on the Autism Spectrum. J Child Psychol Psychiatry. 2014; 55: 374–383. 10.1111/jcpp.12161 24256459 10.1111/jcpp.12161 PMC4532389

[75] Mundy PC, Mastergeorge AM, McIntyre NS. Effect of autism on social learning and social attention In Mundy P. and Mastergeorge A.M. (Eds), Educational Interventions for Students with Autism (p. 3–34). San Francisco CA: Jossey Bass; 2012.

[76] Bodrova E, Leong DJ. Tools of the mind: The vygotskian approach to early childhood education (2nd ed.). Upper Saddle River, NJ: Prentice-Hall; 2007.

[77] Barnett S, Jung K, Yarosz DJ, Thomas J, Hornbeck A, Stechuk R, Burns S. Educational effects of the tools of the mind curriculum: A randomized trial. Early Child Res Quart. 2008; 23: 299–313. 10.1016/j.ecresq.2008.03.001

[78] Horlin C, Falkmer M, Parsons R, Albrecht MA, Falkmer T. The Cost of Autism Spectrum Disorders. PLoS One. 2014 9(9): e106552 10.1371/journal.pone.0106552 25191755 PMC4156354

